# Synthesis of Alkanethiolate-Capped Metal Nanoparticles Using Alkyl Thiosulfate Ligand Precursors: A Method to Generate Promising Reagents for Selective Catalysis

**DOI:** 10.3390/nano8050346

**Published:** 2018-05-18

**Authors:** Khin Aye San, Young-Seok Shon

**Affiliations:** Department of Chemistry and Biochemistry, California State University Long Beach, 1250 Bellflower Blvd., Long Beach, CA 90840, USA; khinsan18@gmail.com

**Keywords:** Bunte salts, alkylthiosulfate, nanoparticles, catalysis, hydrogenation, isomerization, palladium, platinum

## Abstract

Evaluation of metal nanoparticle catalysts functionalized with well-defined thiolate ligands can be potentially important because such systems can provide a spatial control in the reactivity and selectivity of catalysts. A synthetic method utilizing Bunte salts (sodium *S*-alkylthiosulfates) allows the formation of metal nanoparticles (Au, Ag, Pd, Pt, and Ir) capped with alkanethiolate ligands. The catalysis studies on Pd nanoparticles show a strong correlation between the surface ligand structure/composition and the catalytic activity and selectivity for the hydrogenation/isomerization of alkenes, dienes, trienes, and allylic alcohols. The high selectivity of Pd nanoparticles is driven by the controlled electronic properties of the Pd surface limiting the formation of Pd–alkene adducts (or intermediates) necessary for (additional) hydrogenation. The synthesis of water soluble Pd nanoparticles using ω-carboxylate-*S*-alkanethiosulfate salts is successfully achieved and these Pd nanoparticles are examined for the hydrogenation of various unsaturated compounds in both homogeneous and heterogeneous environments. Alkanethiolate-capped Pt nanoparticles are also successfully synthesized and further investigated for the hydrogenation of various alkynes to understand their geometric and electronic surface properties. The high catalytic activity of activated terminal alkynes, but the significantly low activity of internal alkynes and unactivated terminal alkynes, are observed for Pt nanoparticles.

## 1. Introduction

Research on nanomaterials has been popular for more than two decades since they exhibit unique electrical, optical, and physical properties compared to their counterpart bulk materials [1,2,3,4]. Based on synthetic and preparation methods, the nanomaterials of organics, metals, metal oxides, and quantum dots with different sizes ranging from 1 to 100 nm and various shapes from 0 to 3 dimensions (from nanocrystals to nanowires to complex structures) were obtained. These nanomaterials were widely studied upon due to their potential applications in catalysis [5,6,7,8,9], drug delivery [10,11], and optical and electronic devices [12,13]. Two major approaches of synthesizing nanostructures have been a top-down approach such as ion etching and lithography and a bottom-up approach such as chemical/physical vapor deposition and electrochemistry.

Metal nanoparticles which have unique catalytic, electronic, and optical properties such as electron tunneling and surface plasmon resonance have been one of the most studied nanomaterials [1,2,3,4]. They also offered ease in controlled synthesis and surface modification, which allowed tuning their size and shape-dependent properties. The bottom-up approach where the metal ions were reduced by the reducing agent and the nanoparticles were grown in the presence of capping ligands has been widely used. It was found that the type of ligands and applied synthetic conditions could systematically alter their size and ligand–metal ratio and directly influence the chemical and physical properties of metal nanoparticles [14]. The biggest obstacle of using metal nanoparticles for various applications was their tendency to aggregate over time, which leads to a deterioration in their overall activity. The stability of metal nanoparticles against aggregation and oxidation could be adequately enhanced by using organic ligands with a strong affinity to metal nanoparticle surfaces. Different organic compounds containing reactive head groups such as thiol [15,16,17], disulfide [18,19,20], ammonium [21,22,23], amine [24,25,26], and multidentate carboxylate (e.g., citrate) [27,28,29] have been used as protecting ligands. The head group of these ligands interacted with the metal surface and the ligands stabilized the highly reactive surface atoms. The alkyl spacer between the head and the tail groups of the ligand provided a capping shell and controlled the interparticle spacing. In addition, the functional tail groups of the ligand determine the surface reactivity and solubility of the nanoparticles [2]. The type and the degree of ligand capping along with the overall size and shape of nanoparticle core were shown to have a large influence on the optical and electronic properties of metal nanoparticles.

The synthesis of alkanethiolate-protected Au nanoparticles (AuNP) was popularized after the development of the two-phase Brust–Schiffrin method in 1994 [30]. The AuNP produced by this method was highly stable and could be easily isolated [2,14]. The average particle size of these gold nanoparticles ranges from 1 to 3 nm. Based on the high-resolution TEM images, the structure of the nanoparticles was observed to be cuboctahedral and icosahedral. In addition to their potential applications described above, this thiolate-stabilized AuNP has received growing interests from biomedical communities for targeting cancer and drug delivery [31]. In a typical two-phase Brust–Schiffrin reaction, the metal source and the phase transfer agent, tetra-n-octylammonium bromide (TOAB), were mixed until the metal ions were transferred to the organic phase. To the organic layer, a thiol ligand was added to reduce Au(III) to Au(I) and the subsequent addition of a reducing agent, NaBH_4_, reduced Au(I)-SR (gold(I)-alkanethiolate) to form AuNP stabilized by thiolate. The synthesis of monodisperse nanoparticles using the same method could be further extended to different metals such as Ag [32,33], Pd [34,35], and Pt [36]. By utilizing the two-phase synthesis method, the surface of the nanoparticles could be functionalized, and the size and properties of the nanoparticles could be finely tuned with the controlled growth of particles via the ligand-passivation process.

**Scheme 1:**
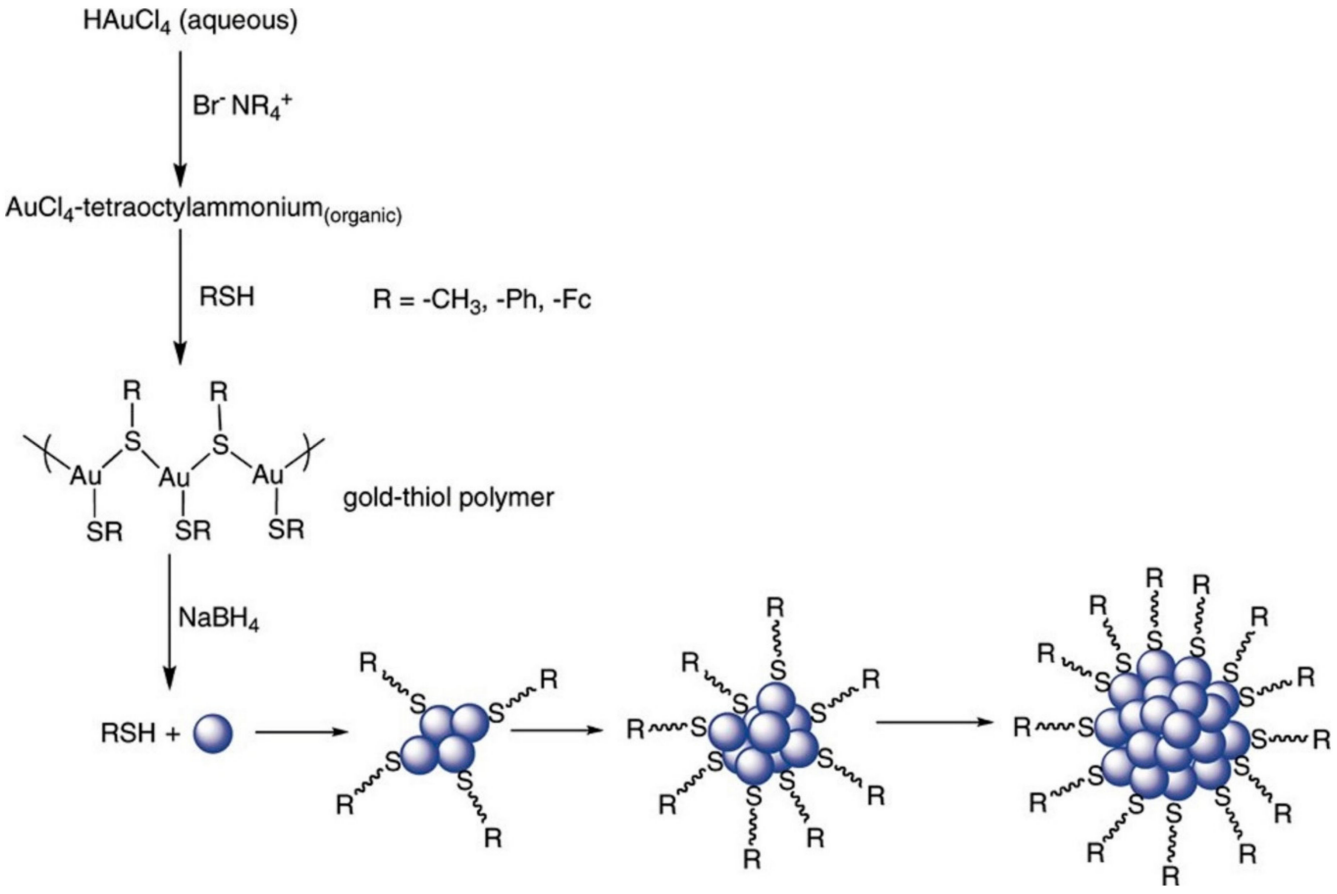
The synthesis of gold nanoparticles by using Brust–Schiffrin method. Reproduced from Ref. [14] with permission from the American Chemical Society, 2009.

The mechanism of the two-phase synthesis of thiolate-stabilized Au nanoparticles has been extensively studied by several groups for better understanding the effects of the reaction conditions and species during the nucleation-growth-passivation process. The factors affecting the core size of the nanoparticles are the reaction conditions such as thiol/Au ratio, temperature, and delivery of NaBH_4_ [37]. Generally, the gold–thiol polymer has been known to be the key intermediate during the reaction (Scheme 1) [37,38,39]. However, Lennox et al. reported that Au^3+^- tetraalkylammonium complexes instead of metal–thiolate species of Au^1+^ are present in the reaction before the reduction by NaBH_4_ [40]. Later, it was found that in the presence of water, Au(I) thiolate species are present as the precursor, whereas in the organic solvent, tetraalkylammonium metal complexes are seen as the main precursor [41,42]. Xue group also found that the reaction intermediate depends on the concentration of the reactants. Their results indicated that gold(I) thiolate species are still formed without the presence of water when the concentration of the reagents is high [43,44]. The presence of [TOA][AuX_2_] (TOA: tetraoctylammonium) species was found to favor the formation of small monodispersed nanoparticles, whereas the large excess of gold(I) thiolate species does not. These mechanistic studies confirmed the importance of understanding the role of the intermediates and the key precursor species present throughout the Brust–Schiffrin two-phase reactions.

Thiols have been widely used as ligands to stabilize and functionalize metal nanoparticles such as gold, silver, copper, palladium, and platinum due to their strong affinity toward the bare metal surfaces. Other sulfur-based compounds such as disulfide [45], sulfide [46,47], thioether [48], and thiosalicylic acid [49] have been utilized as well. The drawback of thiolate-stabilized nanoparticles synthesized using the Brust–Schiffrin method was that the thiol ligands form a densely packed monolayer, which in turn inhibits the catalytic activity of the metal surface [50,51]. Therefore, developing a new strategy to control the surface ligand density of thiolate-capped metal nanoparticles was necessary to set off the interests in the potential application of these nanoparticles as chemo-, regio- and stereo-selective catalysts [52,53].

An alternative capping ligand, alkyl thiosulfate, has recently gained more attention for the synthesis of various metal nanoparticles [54,55,56]. Alkyl thiosulfates are advantageous because of their less pungent odor and relatively high solubility in aqueous environments compared to their corresponding thiols. Alkyl thiosulfates are easily synthesized in high yields by nucleophilic substitution of alkyl halide with thiosulfate ions and purified by simple recrystallization of crude products. Since the thiosulfate group exhibits lower reactivity and slower chemisorption kinetics than the thiol group, alkyl thiosulfate provides an opportunity to control the surface density of the monolayer ligand on metal nanoparticles. The cleavage of the S–SO_3_ bond after the adsorption of alkyl thiosulfates on the surface of metal nanoparticles is also an important characteristic of thiosulfate ligands that allows the formation of a stable thiolate–metal bond. The adsorption of sodium *S*-alkylthiosulfates is known to produce thiolate monolayers on various metal surfaces but is kinetically slower than that of thiols [57,58]. Both the temporary presence of a sulfite moiety on the surface of the metal nanoparticles and the presence of polar thiosulfate functional group of incoming ligand precursors seem to slow the kinetic adsorption of *S*-alkylthiosulfates onto the surface of partially passivated metal nanoparticles with hydrophobic monolayers during the nucleation-growth passivation. The lower surface coverage of alkanethiolate ligands on metal nanoparticle provides controlled poisoning necessary for highly selective catalytic reactions. The first part of this article reviews the characterization and mechanistic studies of various metal nanoparticles synthesized using the thiosulfate protocol. The second part focuses on the catalytic activity and selectivity of alkanethiolate-capped Pd and Pt nanoparticles for hydrogenation and/or isomerization of various unsaturated organic compounds. These main sections are followed by Conclusion and Perspectives with a brief comment on future prospects.

## 2. Synthesis of Metal Nanoparticles Using the Thiosulfate Protocol

The synthesis of thiolate-protected metal nanoparticles such as gold, silver, palladium, platinum, and iridium nanoparticles using the thiosulfate protocol has so far appeared in the literature. This entry is organized into five main sections, each section focusing on one type of metal nanoparticle (Au, Ag, Pd, Pt, and Ir). 

### 2.1. Gold Nanoparticles

Gold nanoparticles (AuNPs) have drawn immense attention since the late 1980s due to their unique size-dependent optical and electronic properties, well-known chemical stability, and impact on a wide range of applications from catalysis, to chemical and biomolecule sensors, and to storage and devices [14]. Much research has focused on using thiols as the capping ligand to stabilize and functionalize AuNPs. However, it is hard to control the surface ligand density since thiol has high reactivity toward the metal surface. By using the thiosulfate ligand, the kinetic adsorption of surface capping ligands on the nanoparticles can be systematically controlled. The examples of thiosulfate ligand capping on the synthesis of AuNPs is summarized in this section.

**Scheme 2:**
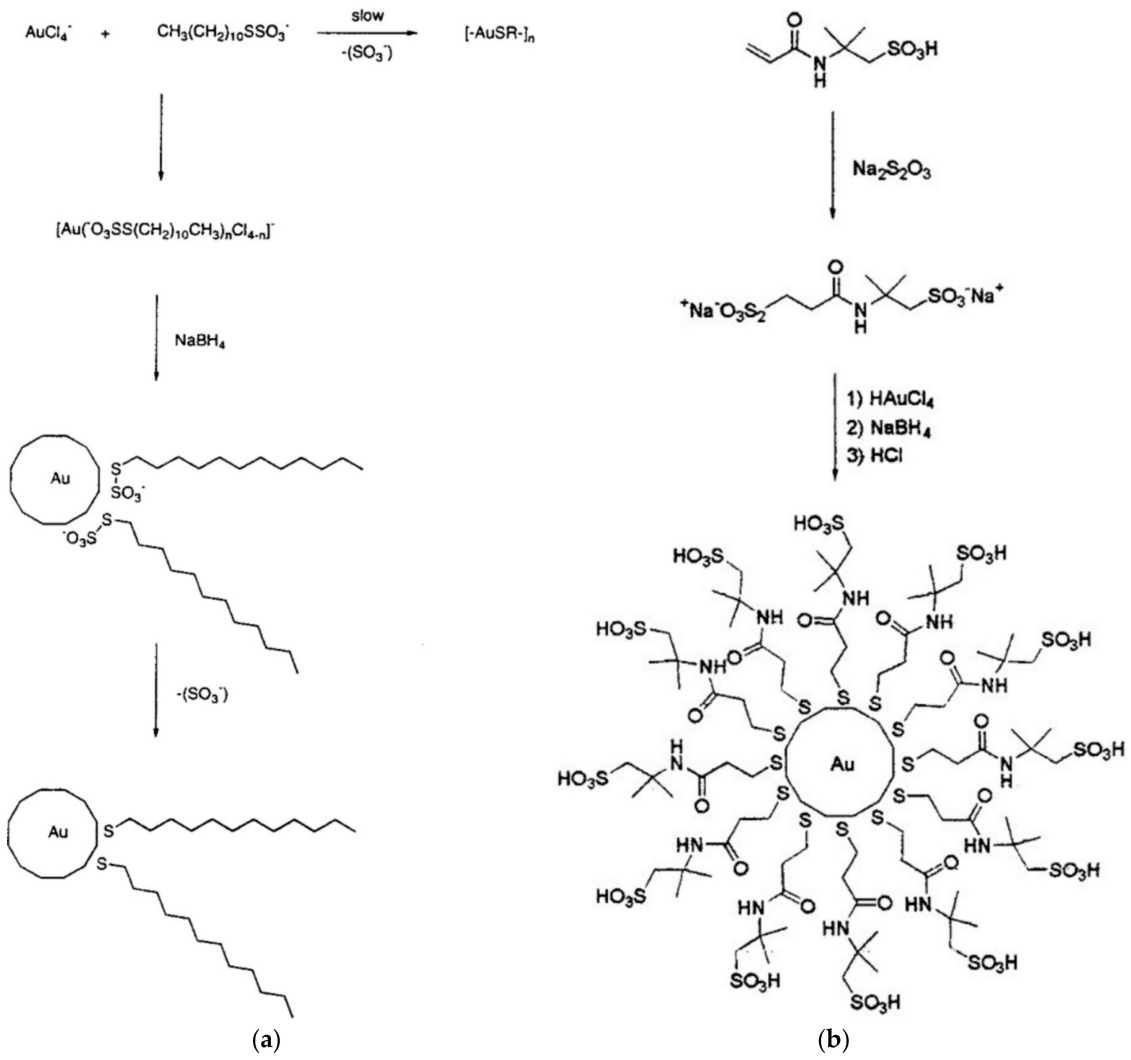
(**a**) Synthesis of AuNP using the S-dodecylthiosulfate ligand and (**b**) synthesis of SO_3_–AuNP using the acid-functionalized thiosulfate ligand. Reproduced from Ref [54] and [55] with permission from the American Chemical Society, 2000 and 2001, respectively.

The first example of metal nanoparticles synthesized using the thiosulfate protocol, which was reported in 2001, was alkanethiolate-protected AuNPs prepared from the sodium S-dodecylthiosulfate ligand [54]. The size and characteristics of these gold nanoparticles have been directly compared to those formed from the dodecanethiol ligand under the same condition. The reaction pathway for the synthesis of dodecanethiolate-protected AuNPs by using the thiosulfate protocol is shown in Scheme 2a. After the thiosulfate ligand has been adsorbed onto the surface of gold, the sulfite group was eliminated from the surface as proved by X-ray photoelectron spectroscopy (XPS) studies. The results also showed that the thiolate-protected AuNPs generated from S-dodecylthiosulfate have a larger particle size (3.6 ± 2.3 nm) compared to those synthesized from dodecanethiol (2.2 ± 1.2 nm). The UV-Vis spectra of AuNPs generated from S-dodecylthiosulfate also showed a larger surface plasmon band of gold at ~520 nm due to the increased average size. The increase in particle size indicated that the passivation kinetics of the thiosulfate ligand is slower than that of the thiol ligand. The molar concentration of the ligand is known to control the average core sizes of the AuNPs synthesized from the Brust–Schiffrin method using alkanethiols. The thiosulfate protocol studies also showed that an increase in the molar ratio of the thiosulfate ligand to Au from 2 to 3 slightly decreases the core size of AuNP. The change in the reaction time between the thiosulfate ligand and the Au precursor before the addition of the reducing agent did not affect the size of nanoparticles. The absence of any color change upon the addition of the thiosulfate ligand indicated that the reduction of Au^3+^ to Au^1+^ does not occur or take place very slowly, which was clearly different from the Brust–Schiffrin method. A different solvent system (THF/H_2_O) that would not require a phase transfer agent during the synthesis produced AuNPs with a smaller average core size compared to the original thiosulfate system using the biphasic toluene/H_2_O solvent. The reason for this faster passivation in THF/H_2_O, which was later discovered during the synthesis of Pd nanoparticles from thiosulfate in THF/H_2_O, is the formation of thiol from thiosulfate by NaBH_4_-induced hydrolysis in THF/H_2_O [59].

Functional groups could be incorporated using the thiosulfate protocol into the monolayer to yield nanoparticles with specific properties and chemical reactivity. Water-soluble monolayer-protected nanoparticles (SO_3_–AuNP) have been prepared by using the strong acid-functionalized thiosulfate ligand of 2-acrylamido-2-methyl-1-propanesulfonic acid [55]. The reaction pathway is shown in Scheme 2b. The average core diameter of these synthesized nanoparticles was 2.2 ± 1.1 nm (ligand/gold ratio = 3), which was smaller than those synthesized from the S-dodecylthiosulfate ligand. The SO_3_–AuNP had a monolayer thickness of ~1.4 nm and 30% organic weight fraction. Since SO_3_–AuNP had a strong acidic property, it could be utilized in acid/base titrations. Titration results showed that SO_3_–AuNP is a slightly weaker acid than the corresponding free acidic ligand, 2-acrylamido-2-methyl-1-propanesulfonic acid. The anionic nature of SO_3_–AuNP allowed the material to be further modified to ionically conductive materials by neutralizing SO_3_–AuNP with polyether-tailed triethylammonium hydroxide and tetrabutylammonium hydroxide. These quaternary ammonium salts of water-soluble nanoparticles could act as ionically conductive molten salt (or ionic liquid).

Further controlling the core size and ligand structure of thiolate-capped AuNPs using the thiosulfate protocol has been extensively studied by Hutchinson et al., who focused on the synthesis of functionalized AuNPs with a wide range of core diameters from 1.5 to 20 nm [60]. This is another advantage of the thiosulfate protocol compared to the thiol-based Brust–Schiffrin reaction, which is limited to the generation of gold nanoparticles with core sizes ranging from 1 to 5 nm. By using thiosulfate ligands, which have weaker interactions with nanoparticle surfaces and slower passivation kinetics due to the polar head group, the resulting nanoparticles could be grown into larger particles during the nucleation-growth-passivation process. The Hutchinson group systematically optimized the reaction conditions (ligand/gold ratio, reaction temperature, and reducing agent concentration) to produce various sizes and with different functionality, including hydroxyl, carboxylate, and quaternary ammonium ligands. The results showed that the lower ligand/gold ratio and higher temperature yielded larger particles with core sizes up to 20 nm in diameter (Table 1). At lower temperatures, the ligand shell adsorbed strongly to the metal surface and decreased the growth rate, resulting in smaller particles. The higher concentration of the reducing agent, sodium borohydride, yielded smaller nanoparticles, since the increased formation of small seed particles limited the amount of the metal precursor necessary for further particle growth. The concentration of the reducing agent also determined the polydispersity of the nanoparticles, resulting in higher polydispersity when the lower concentration of the reducing agent was applied. Table 1 lists the range of various particle sizes produced by using the thiosulfate (Bunte salt: BS) of the mercaptoethoxyethoxyethanol ligand (MEEE). The results confirmed that monolayer-protected AuNPs could be synthesized with a larger core diameter by utilizing the thiosulfate ligand compared to those synthesized using thiol ligands.

**Scheme 3:**
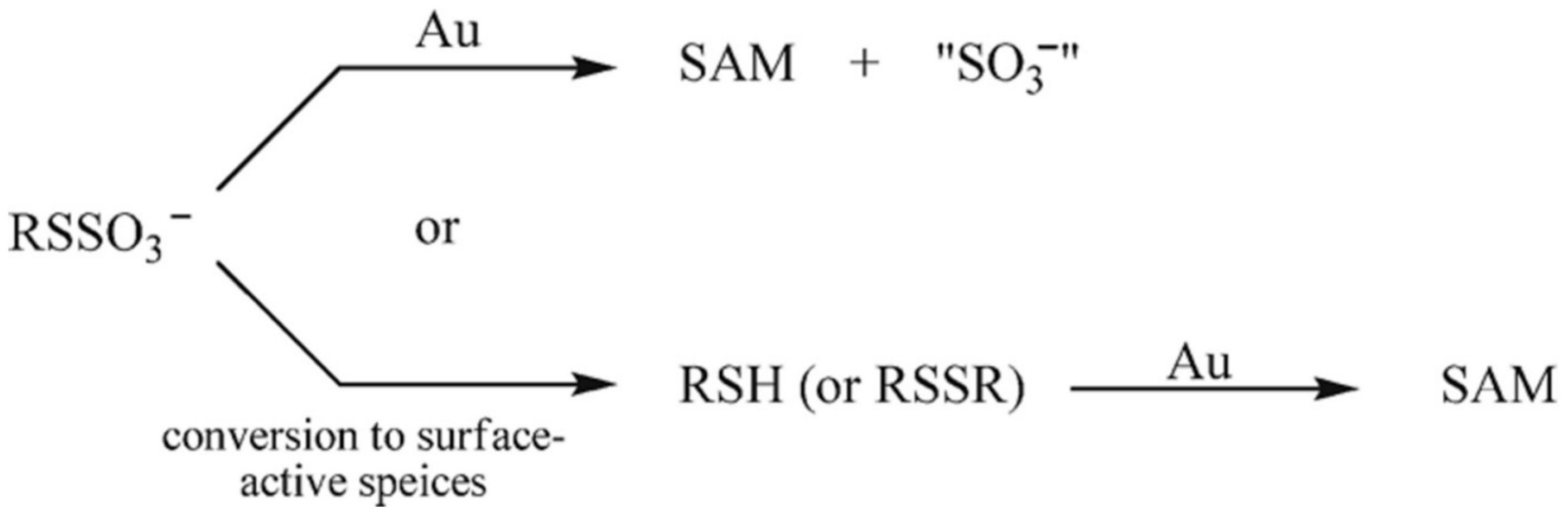
The mechanism of self-assembled monolayer formation on gold surface. Reproduced from Ref [58] with permission from the American Chemical Society, 2011.

In addition to stabilizing AuNPs, these alkyl thiosulfate ligands have been utilized to form self-assembled monolayers (SAMs) on the flat gold surface. SAMs have been popularly used for the protection of the bare metal, metal oxide, or semiconductor surfaces from corrosion or oxidation. SAMs have also been used for the functionalization of nanostructure surfaces for specifically targeted applications [2]. The Ferguson group studied how functionalized thiosulfates could form a monolayer on the gold surface by using an electrochemical approach [57]. The wide range of functionalized alkyl thiosulfates, including terminal methyl, fluorinated alkyl, carboxylic acid, ester, amide, hydroxyl, and vinyl groups, have been tested. The results showed that the SAMs with slightly lower packing density and lower ellipsometric thickness were formed on gold electrodes compared to those of thiols. The slower kinetics of alkyl thiosulfates were the exact cause for the formation of the less dense monolayer. The Ferguson group also studied the mechanism of spontaneous adsorption of alkyl thiosulfate on gold substrate (Scheme 3) [58]. Studies showed that the direct adsorption of alkyl thiosulfate on gold substrate does not occur spontaneously. Instead, controlled experiments under the electrochemical condition proved that the hydrolysis reaction of alkyl thiosulfate followed by the self-assembly of the resulting alkanethiol or disulfide is the main mechanism. When oxidative potential was applied to the targeted gold electrode, the reduction of the S–SO_3_^−^ bond occurred and the self-assembly of ligands was then initiated on the gold surface. These results of the assembly of alkyl thiosulfate on flat gold surfaces under electrochemical conditions suggested that the self-assembly of thiosulfate ligands on spherical AuNPs might be catalyzed by the surface-adsorbed borohydride reducing the S–SO_3_^−^ bond after the physisorption of thiosulfate ligands on nanoparticle surfaces. Further investigation on the formation of thiolate monolayers from alkyl thiosulfates on nanoparticle surfaces would be necessary for the complete understanding of thiosulfate ligand capping processes.

### 2.2. Silver Nanoparticles

**Scheme 4:**
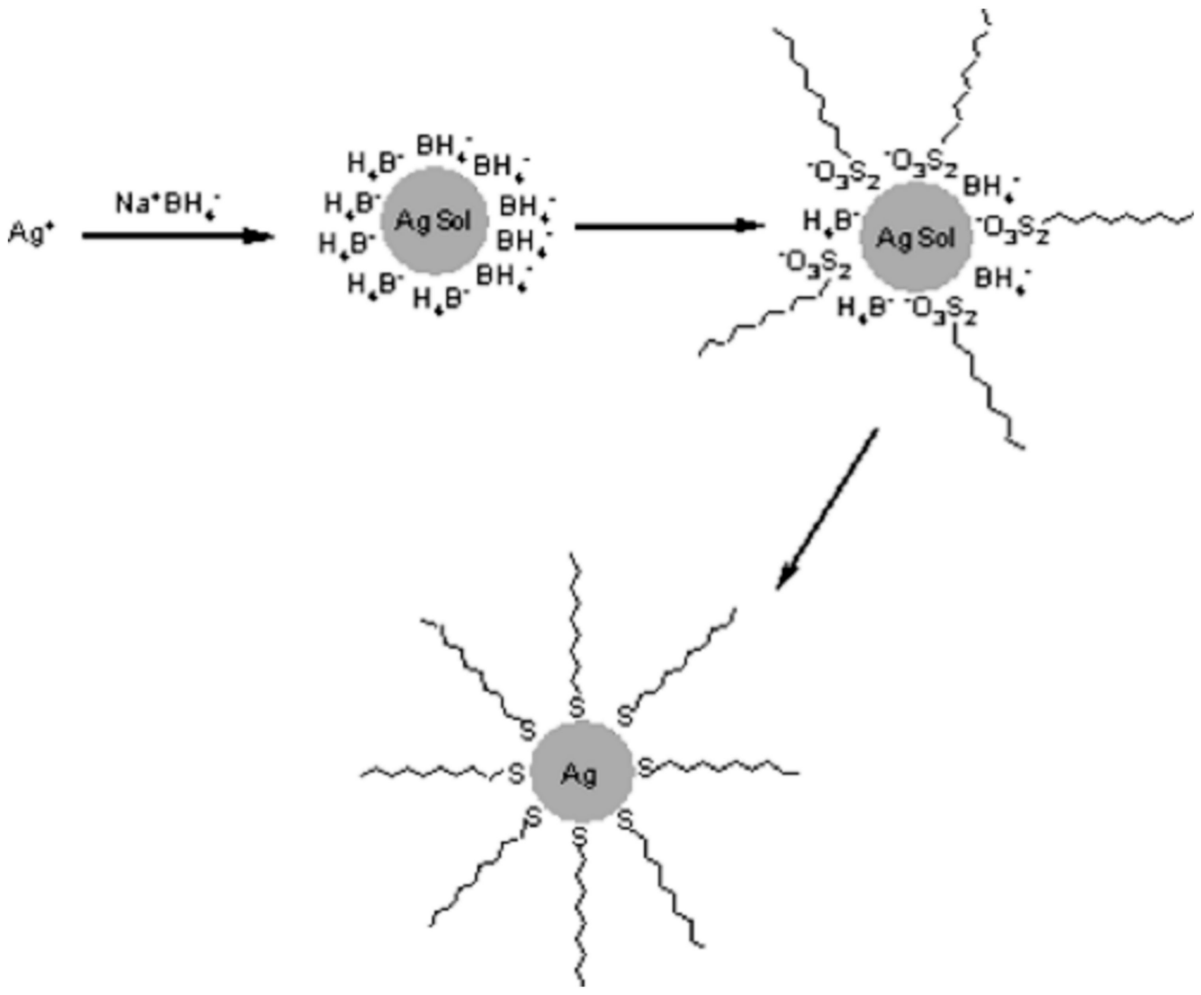
Synthesis of AgNPs using S-dodecylthiosulfate in H_2_O. Reproduced from Ref. [56] with permission from the American Chemical Society, 2004.

Silver nanoparticles (AgNPs) have drawn interest due to their unique optical and antimicrobial properties and their applications in surface-enhanced Raman spectroscopy [3]. The thiosulfate protocol could be used for the preparation of alkanethiolate-capped AgNPs in both single-phase (H_2_O) and two-phase (H_2_O/Toluene) systems [55]. Single-phase synthesis of AgNPs using water as the single solvent is considerably greener and less expensive compared with the two-phase method. Scheme 4 shows the reaction pathway for the synthesis of alkanethiolate-protected AgNPs in water. The aqueous silver nitrate precursor was first reduced by sodium borohydride and small silver clusters (Ag sols) were formed. These silver sols were stabilized by borohydride and borate, which is the hydrolysis product of borohydride, forming an electrostatic layer that prevents particle aggregation. Later, an S-dodecylthiosulfate ligand was added and adsorbed onto the surface, forming thiolate-stabilized AgNPs. The single-phase synthesis of AgNPs from dodecanethiol was ineffective in producing stable colloidal AgNPs due to the insolubility of dodecanethiol in water.

The synthesized AgNP in water using the thiosulfate protocol was compared to the AgNP synthesized from the two-phase system (H_2_O/Toluene). In single-phase synthesis, Ag–thiolate complexes were formed in addition to stable AgNP at room temperature, which was evidenced by UV-Vis spectra of AgNP showing an absorption band at 350 nm. Ag–thiolate complexes were formed by the oxidation of AgNP during and after the nanoparticle formation. However, when the reaction temperature was increased to 50 °C, the absorption band for the Ag–thiolate complex almost disappeared in the UV-Vis spectra. The core sizes of AgNP at both temperatures were almost consistent, as shown in Table 2. The AgNPs synthesized from either dodecanethiol or S-dodecylthiosulfate in the two-phase system (H_2_O/toluene) were highly stable and prevented from oxidation. The core size of the AgNP generated from alkyl thiosulfate in the two-phase system was slightly larger than those of corresponding AgNPs synthesized in single phase and prepared using the Brust–Schiffrin reaction. This shows that the passivation kinetics of alkyl thiosulfates in two phases is slower than that of the single-phase system and that of alkanethiols in the two-phase system.

Suber et al. performed further optimization studies on the synthesis of AgNP in a single-phase thiosulfate system since the previous method described above resulted in a relatively low yield of AgNP with the formation of Ag–thiolate by-products [61]. By reducing or hindering the formation of the Ag–thiolate complex, the purification step and low particle yields could be avoided. By varying the reaction condition, they were able to obtain stable and pure alkanethiolate-stabilized AgNPs. Instead of adding a thiosulfate ligand after the addition of a reducing agent, the stabilizing ligand was added before the reducing agent (NaBH_4_) to prevent the initial particle aggregation. In addition to adding NaBH_4_, the antioxidant agent ascorbic acid was added to inhibit the oxidation of Ag^0^. The addition of ascorbic acid made the solution reductive and successfully prevented further oxidation of Ag particles. The solvent system was also changed from water to ethanol to assist the facile product separation. The core size of the nanoparticles was dependent upon the molar ratio of ligand-to-metal precursor (L/M). By decreasing the molar ratio of L/M from 2 to 0.75, the particle size increased from 3.0 to 4.0 nm. With this reported optimized synthesis condition, alkanethiolate-capped AgNPs could be obtained in a pure form using the thiosulfate protocol and the core sizes could be tailored by changing the molar concentration of ligand.

### 2.3. Palladium Nanoparticles

**Scheme 5:**
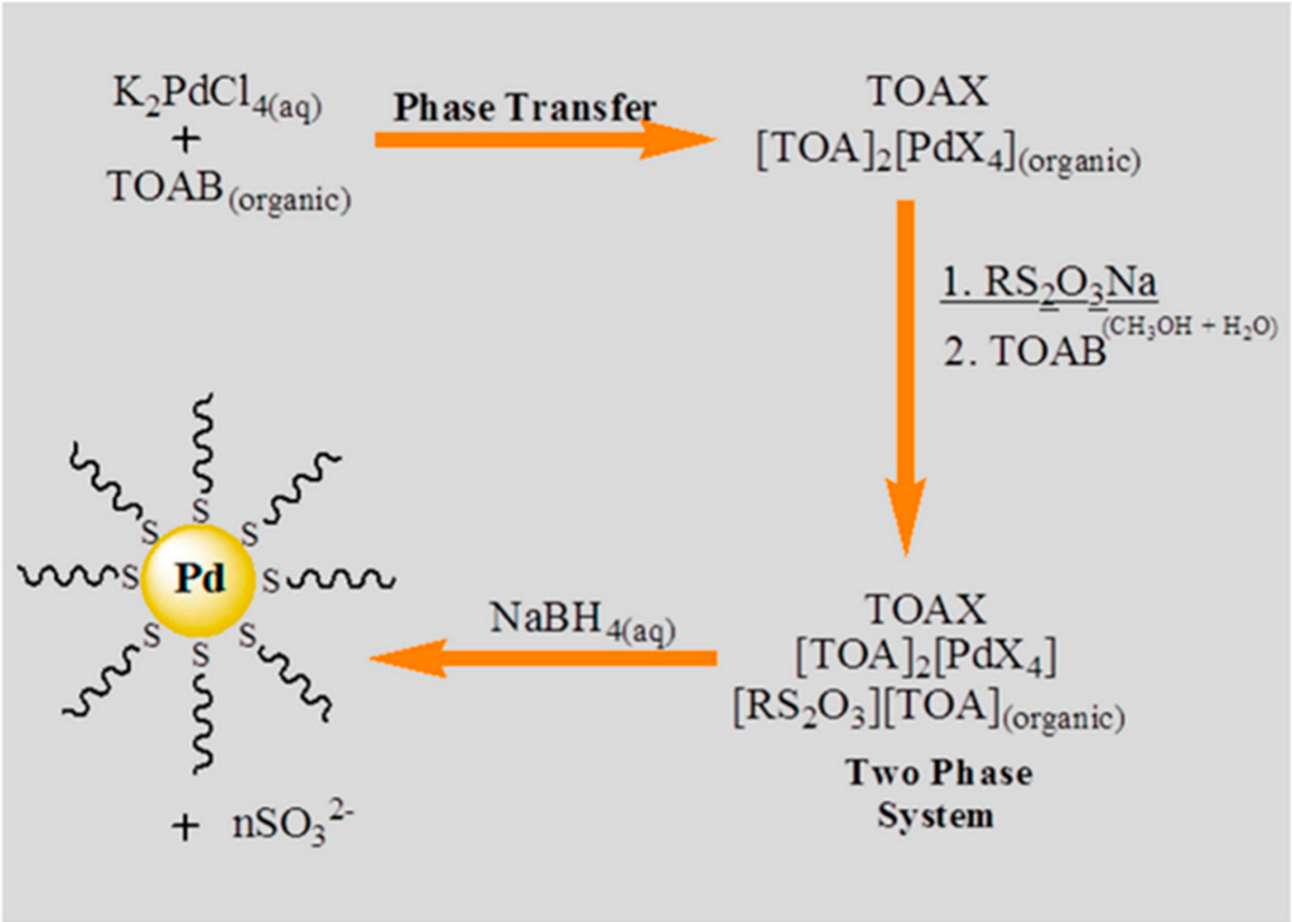
Synthesis of alkanethiolate-capped PdNP generated from S-dodecylthiosulfate. Reproduced from Ref. [59] with permission from the American Chemical Society, 2012.

The thiosulfate protocol for catalytic PdNP synthesis required alkyl thiosulfate ligands in a two-phase system to prevent the hydrolysis of the thiosulfate group [59,62]. The sodium S-dodecylthiosulfate ligand was therefore introduced to the organic layer of the Pd precursor with the aid of a phase transfer agent TOAB. PdNP was generated after the addition of the reducing agent NaBH_4_, as described in Scheme 5. The mechanistic studies for the thiosulfate protocol indicated that it was different from the reported mechanism of AuNP synthesis using the thiol ligand. For the Brust–Schiffrin reaction using thiol ligands, the reduction of the Au precursor and the oxidation of thiol to disulfide were observed. However, the addition of the thiosulfate ligand to the Pd precursor did not accompany similar chemical reactions and instead resulted in the formation of the [TOA]_2_[PdX_4_] complex. The direct reduction of [TOA]_2_[PdX_4_] by NaBH_4_ produced PdNP with the average core diameter of 2–3 nm and the organic content of ~30%. The surface ligand density of PdNPs generated from the thiosulfate ligand was lower than that of the PdNPs synthesized from thiol due to the slower passivation kinetics of the thiosulfate ligand [62].

Further investigation showed that the core size and ligand density of PdNPs can be controlled by varying synthetic conditions such as the molar equivalent of ligand, TOAB, and NaBH_4_ and the reaction temperature [59]. The explored synthetic conditions are summarized in Table 3, in which the numbers represent the molar equivalent of each reaction species to one equivalent of Pd precursor. The resulting surface ligand coverage and core diameters of PdNPs from each reaction condition indicated that an increase in the reaction temperature (i → ii) yields NP with a smaller core size and higher surface ligand density. By decreasing the molar equivalent of the phase transfer agent (i → iii) TOAB, the NP core diameter decreases, and the surface ligand coverage increases. This indicated that excess TOAB, which has a role of transferring metal and ligand precursors from the aqueous phase to the organic phase, hinders the passivation by alkyl thiosulfate and is necessary for the generation of NP with lower surface ligand coverage. When the molar equivalent of the ligand was decreased by half (i → iv), the NP core diameter slightly increased, and the surface ligand density decreased. When the molar equivalent of NaBH_4_ was decreased (i → v), the NP core diameter increased, and the surface ligand density remained mostly same. The increase in core diameter was due to the lower concentration of Pd seeds, allowing for the continued growth of nanoparticles.

Utilizing water as the solvent media is more favorable and greener compared to using toxic organic solvents such as toluene and chloroform. The availability of stable and water-soluble colloidal nanoparticle systems is necessary for water-based homogeneous green catalysis of nanoparticles. Water-soluble Pd nanoparticles have been synthesized using ω-carboxyl-S-alkanethiosulfate as the ligand precursor (ligand/palladium molar ratio = 2) in two-phase synthesis [63]. Synthetic conditions were systematically varied to control the core size and ligand density of PdNP. By reducing the amount of the reducing agent NaBH_4_, the core diameter of PdNP slightly increased and the surface ligand coverage decreased. A decrease in the ligand coverage was explained by the presence of fewer TOA^+-^BH_4_ complexes, which resulted in increased availability of TOA^+^ for competing with the thiosulfate ligand during the passivation process. When the chain length of the hydrophilic thiosulfate ligand was decreased, the surface ligand density also decreased because of the higher polarity of shorter alkyl thiosulfate and the increased solubility in the aqueous layer. This characteristic of shorter polar ligand resulted in the lower concentration of thiosulfate ligand in the organic layer and the decreased surface ligand coverage. 

### 2.4. Platinum Nanoparticles

**Figure 1:**
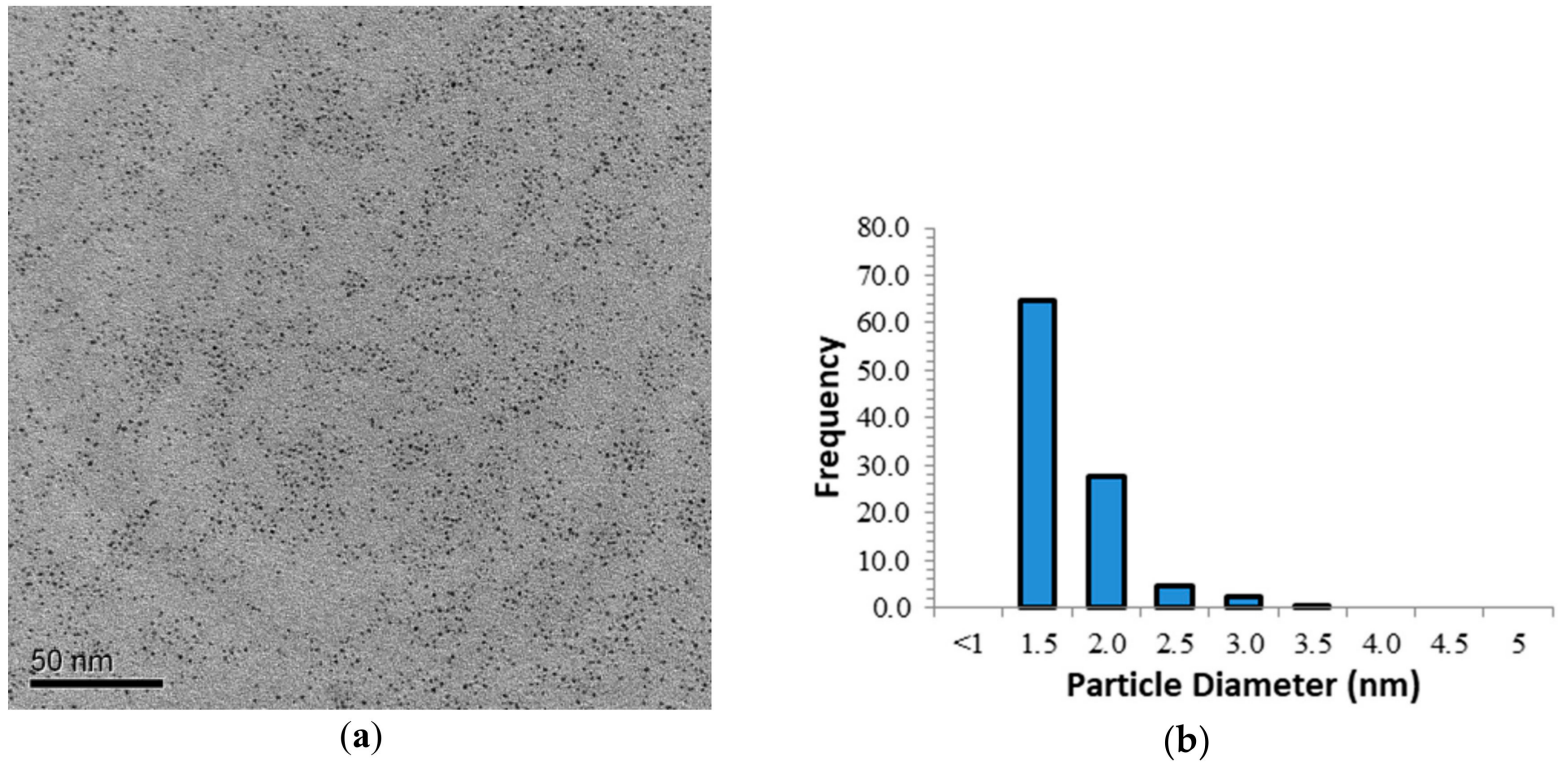
(**a**) TEM image and (**b**) size distribution histogram of PtNP generated from sodium S-octylthiosulfate. Reproduced from Ref. [64] with permission from the American Chemical Society, 2017.

**Scheme 6:**
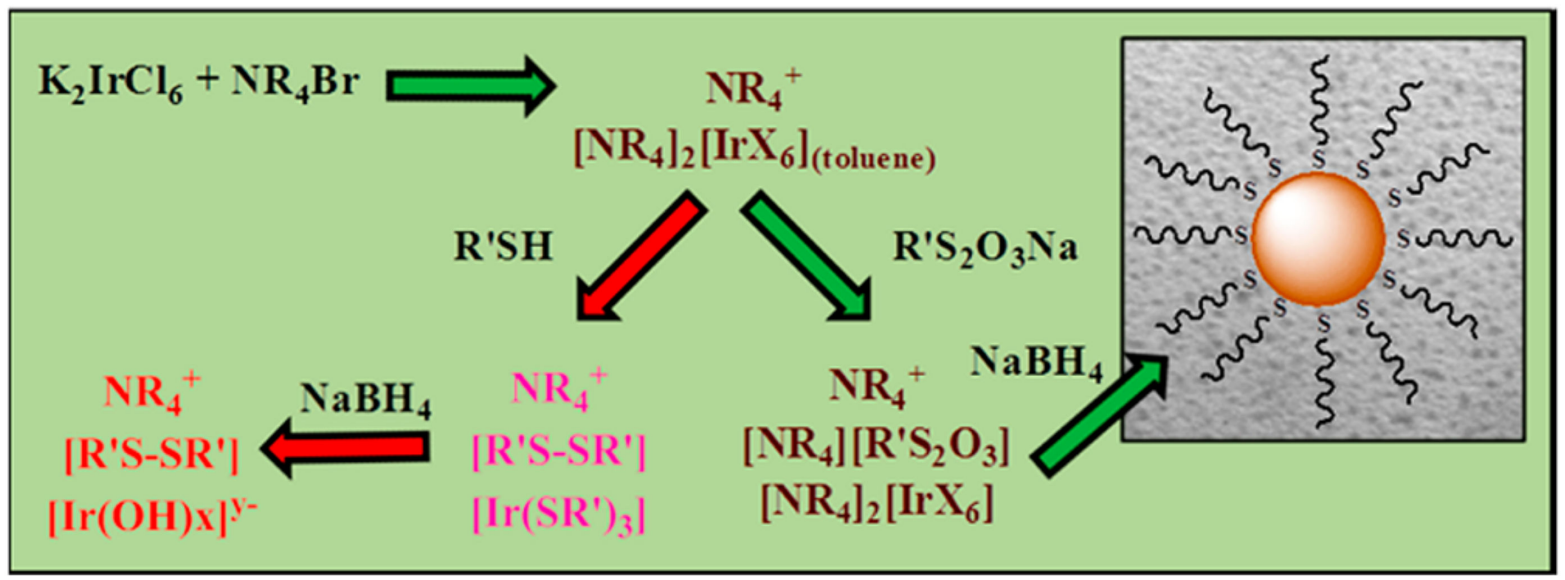
Proposed mechanism for iridium nanoparticles from dodecanethiol and S-dodecylthiosulfate. Reproduced from Ref. [67] with permission from the American Chemical Society, 2014.

Platinum nanoparticles (PtNPs) were synthesized from dihydrogen hexachloroplatinate (IV) (H_2_PtCl_6_.6H_2_O) using the two-phase thiosulfate method similar to Scheme 6 [64]. The mole ratio of thiosulfate ligand to Pt salts was varied from 2:1 to 4:1 for the nanoparticle synthesis. The synthesis with the less amount of thiosulfate ligand failed to produce stable thiolate-capped PtNP. As the mole equivalents of S-alkylthiosulfate ligand was increased, the yield of the alkanethiolate-capped PtNP increased. When PtNP was synthesized in dichloromethane (CH_2_Cl_2_) instead of toluene, the yield for PtNP increased by ~50% compared to that in toluene solvent. The results indicated that the surface passivation activity of alkylthiosulfate might be higher in CH_2_Cl_2_ than in toluene. When the chain length of the ligand was increased from S-octylthiosulfate to S-dodecylthiosulfate, the longer chain length of surface ligand seemed to provide an enhanced protection of the PtNP surface and resulted in a higher yield. Effects on the core size (1.5–2.0 nm) and surface ligand density of PtNP by different reaction conditions stated above were mostly consistent with the expected trends, but with rather small changes. The representative TEM image and histogram of PtNP are shown in Figure 1.

The reaction species formed during the PtNP syntheses for both S-octylthiosulfate and 1-octanethiol were monitored by ^1^H NMR and UV-Vis spectroscopies. When thiol was added to the organic layer of [TOA^+^]_2_[PtCl_6_^2-^], a total of four equivalents of the thiol ligand was necessary for the reduction of Pt^4+^ to Pt^2+^ and the formation of Pt(SR)_2_. After the addition of NaBH_4_, the NMR data showed that Pt(SR)_2_ was still present in the reaction mixture, suggesting an incomplete reduction by NaBH_4_ or a fast decomposition of PtNP-to-Pt complexes such as the Pt(SR)_2_ species [65,66]. When the thiosulfate was added to the organic layer of [TOA^+^]_2_[PtCl_6_^2−^], the formation of a platinum–thiolate complex was not observed. Instead, the intercalation/interaction of S-octylthiosulfate to/with [TOA^+^]_2_[PtCl_6_^2−^] was evidenced by the slight upfield shift of the α-CH_2_-N peak. The reduction of Pt^4+^ by NaBH_4_ successfully produced stable PtNPs.

### 2.5. Iridium Nanoparticles

Stable and isolable iridium nanoparticles (IrNP) could be synthesized from the thiosulfate protocol by using S-dodecylthiosulfate as the ligand precursor [67]. The successful synthesis could only be achieved when the reaction was carried out at a higher temperature of 60 °C. Based on the TEM images, the synthesized IrNP had the average core diameter of 1.2 ± 0.3 nm.

The mechanism of the IrNP formation was also investigated with a focus on understanding the difference between the activities of S-alkylthiosulfate and alkanethiol ligands. The study explored the reaction species throughout by monitoring both synthetic reactions using ^1^H NMR and UV-Vis spectroscopies. When the Ir precursor in aqueous solution was transferred to the organic phase by the phase transfer agent TOAB, the phased-transferred Ir complex and tetraoctylammonium salts maintained an ionic pair in the organic phase. The addition of stabilizing ligands, either dodecanethiol or S-dodecylthiosulfate, to the reaction mixture showed that there was a clear difference between the reactivities of two ligands. The addition of four equivalents of dodecanethiol revealed the formation of the Ir(SR)_3_ complex, since one equivalent of thiol reduced Ir^4+^ to Ir^3+^ and the remaining three equivalents of dodecanethiol formed thiolate species. The addition of NaBH_4_ was not able to reduce Ir–thiolate species and instead the formation of hydrolysis product was observed at the end of reaction. The addition of the S-dodecylthiosulfate ligand to the reaction mixture and the subsequent addition of NaBH_4_ reduced the Ir^4+^ ions to form Ir nanoparticles. The detailed mechanistic studies showed the efficiency of utilizing the thiosulfate ligand in contrast to the thiol ligand in the formation of thiolate-stabilized IrNPs. These IrNPs exhibited relatively strong magnetic properties.

## 3. Catalysis of Metal Nanoparticles Generated by the Thiosulfate Protocol

Ligand-capped palladium and platinum nanoparticles have gained intense research interest since they exhibit high catalytic activity and the surrounding ligands influence catalytic selectivity similar to organometallic catalysts [68,69]. Especially, alkanethiolate-capped PdNP and PtNP catalysts have been used for organic reactions such as hydrogenation and carbon–carbon coupling reactions [53]. The thiosulfate protocol broadened the application of alkanethiolate-capped PdNP and PtNP by increasing the overall activity of catalysts [62]. Functionalized thiolate-protected PdNPs synthesized using the thiosulfate protocol allowed more systematic investigations regarding the effects of ligand functionality and structure in addition to particle sizes and ligand density [59,63,64].

### 3.1. Catalysis of Palladium Nanoparticles

**Scheme 7:**
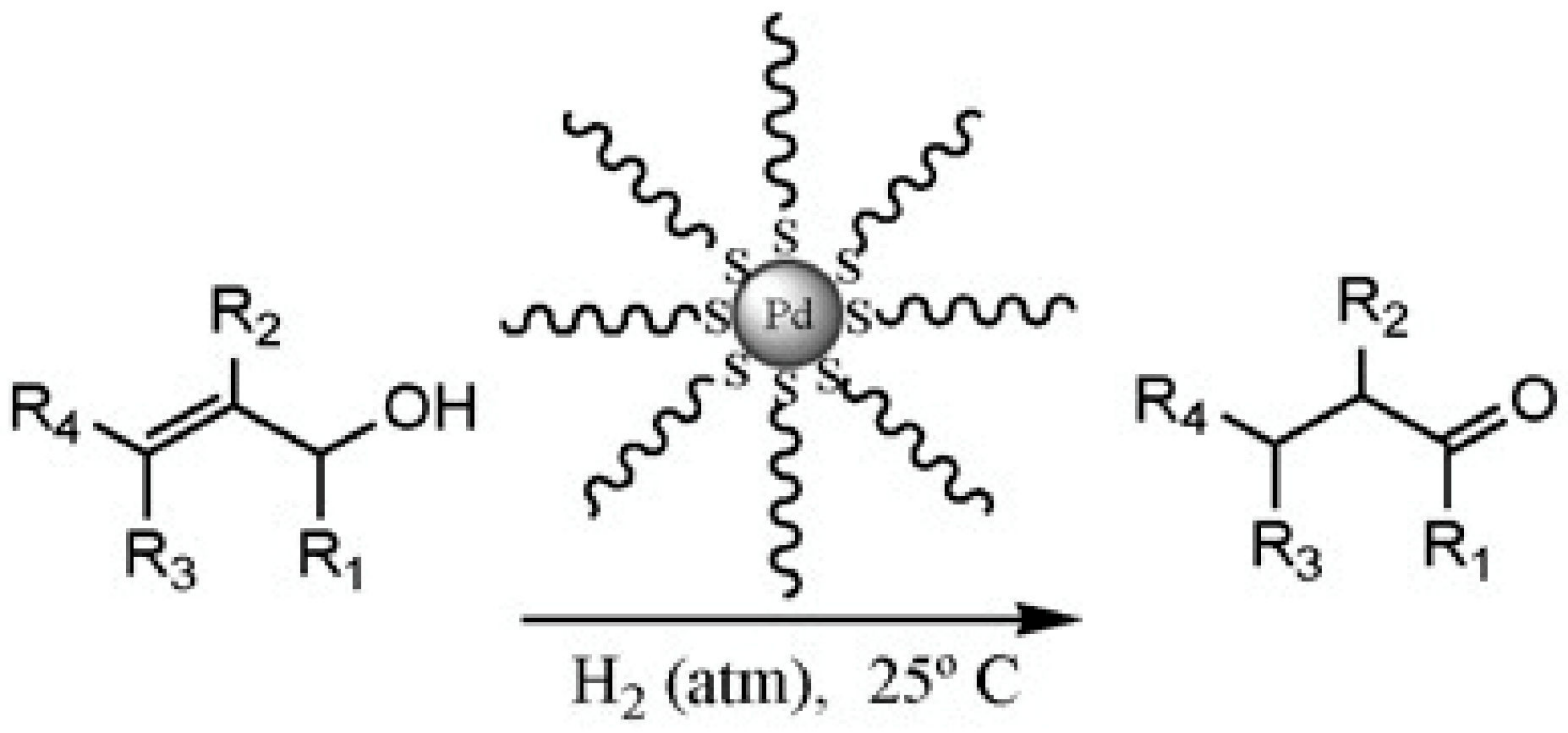
The isomerization of allylic alcohols using synthesized PdNP (5 mol % Pd/substrate, 2.9 mmol substrate in 5 mL CDCl_3_, 1 atm H_2_). R_1-4_ = H, CH_3_, or alkyl. Adapted from Ref. [70] with permission from the Elsevier, 2011.

The catalytic activity of PdNP synthesized from an S-dodecylthiosulfate ligand was examined by the isomerization of allyl alcohol to the corresponding propanal product. The results indicated that the catalytic activity and selectivity of PdNP synthesized from the thiosulfate ligand is much higher than those of PdNP produced from the thiol ligand. Catalytic isomerization of various substituted allylic alcohols was also studied (Scheme 7). The results indicated that isomerization of allylic alcohols via enol intermediate and higher catalytic activity are observed for the substrates that yield more thermodynamically stable enol intermediate. The isomerization of less substituted allylic alcohols resulted in overall higher catalytic activity and selectivity, indicating the presence of the steric interaction between the surface ligands and more substituted substrates. The dodecanethiolate-capped PdNP synthesized using the thiosulfate protocol maintained good stability during the isomerization reaction and could be recycled 14 times without losing the overall catalytic activity and selectivity [70,71].

**Figure 2:**
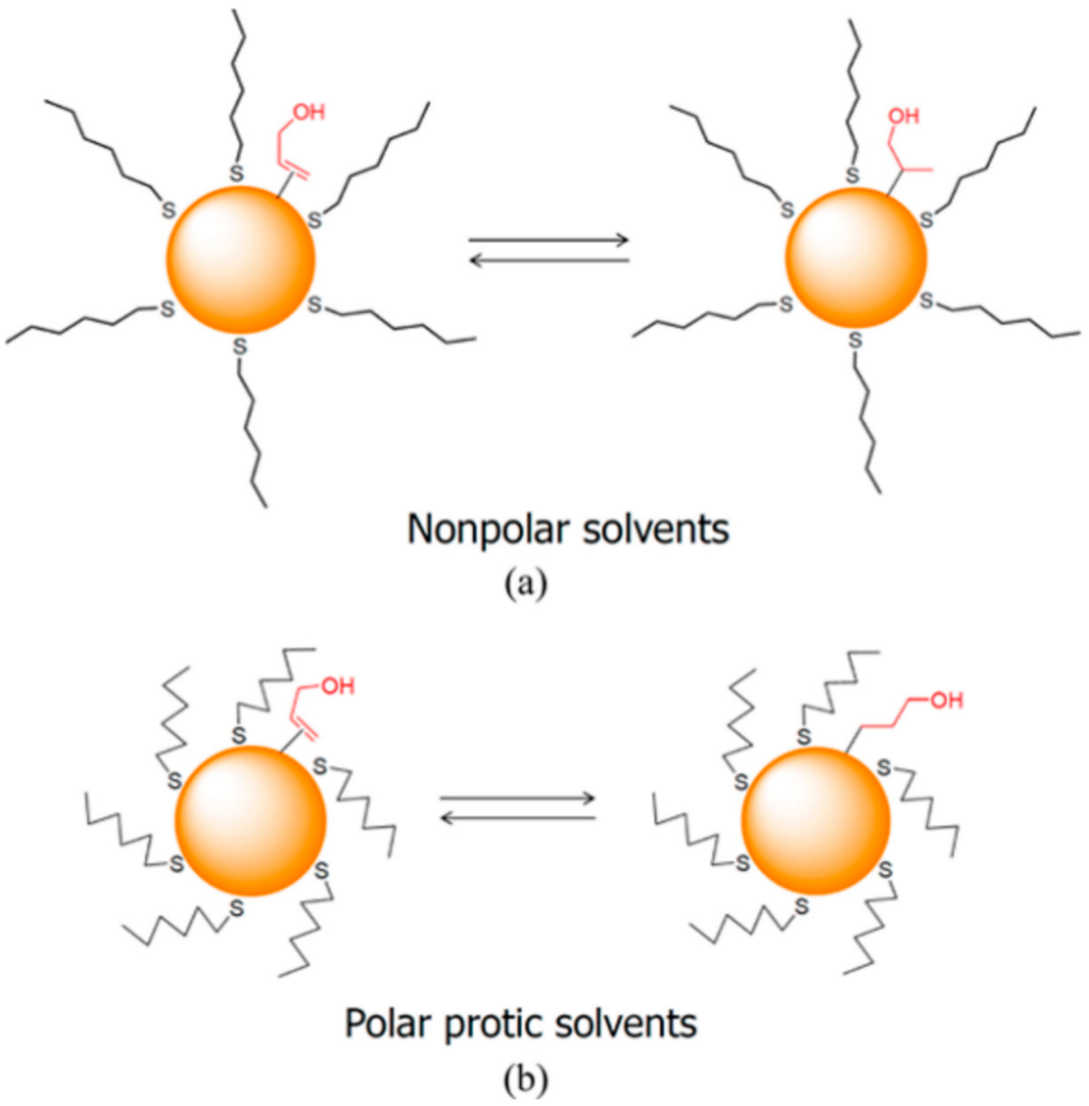
Isomerization and hydrogenation of allyl alcohol in nonpolar (**a**) and polar (**b**) solvents. Reproduced from Ref. [71] with permission from the American Chemical Society, 2012.

Further catalytic studies showed that the optimized stable PdNPs with lower ligand density and larger core sizes (~3 nm) exhibit higher reactivity and selectivity toward the isomerization of allylic alcohols [70]. The isomerization of allyl alcohol has also been studied under various solvents using alkanethiolate-capped PdNP [71]. Thiolate-capped PdNP has nonpolar alkyl chains, which allow PdNP to be homogeneously mixed in less polar solvents such as C_6_H_6_ and CHCl_3_ but make them insoluble in polar protic solvents such as methanol and water. The key intermediate for isomerization and hydrogenation of allyl alcohol is the Pd–alkyl intermediate. It was proposed that branched Pd–alkyl intermediate favors isomerization and linear Pd–alkyl intermediate promotes hydrogenation. The ligand structure and conformation of alkanethiolate ligands on PdNP depended on the polarity of the reaction solvent, as shown in Figure 2. The Pd hydride was added to C=C bond of allyl alcohol to form branched Pd–alkyl intermediate in less polar solvents (Figure 2a). Therefore, the catalytic activity was highly selective toward the isomerization of allyl alcohol than hydrogenation process. In polar protic solvents, the ligand structure was interdigitated and the nanoparticles were aggregated. Thus, the addition of Pd–H to the C=C bond produced a less steric linear Pd–alkyl intermediate (Figure 2b), which resulted in selective hydrogenation. The electronic influence of polar solvents increasing the adsorption of allyl alcohol could be also considered as an additional contributing factor for the altered selectivity of PdNPs toward hydrogenation. The chain length of the ligand was also varied and the results showed that the ligand with a shorter chain length yields PdNP with higher ligand density, leading to a decrease in catalytic activity. The spatial control of the nanoparticle was found to be important in tuning the reactivity and selectivity of the catalyst based on these investigations.

The catalytic activity of water-soluble PdNPs was studied by monitoring the isomerization/hydrogenation of allyl alcohol in both water and chloroform. The catalytic activity was higher for PdNP with lower surface ligand density as described previously. The synthesized water-soluble PdNP dissolved homogeneously in D_2_O showed good catalytic selectivity toward the hydrogenation of allyl alcohol to 1-propanol, whereas the heterogeneous catalysis of PdNP in CDCl_3_ resulted in facilitating the formation of the isomerization product, propanal. The catalytic results indicated that the solvent polarity dependent ligand conformation and/or surface electronic property are important in controlling the catalytic activity and selectivity of the PdNP as described above.

**Figure 3:**
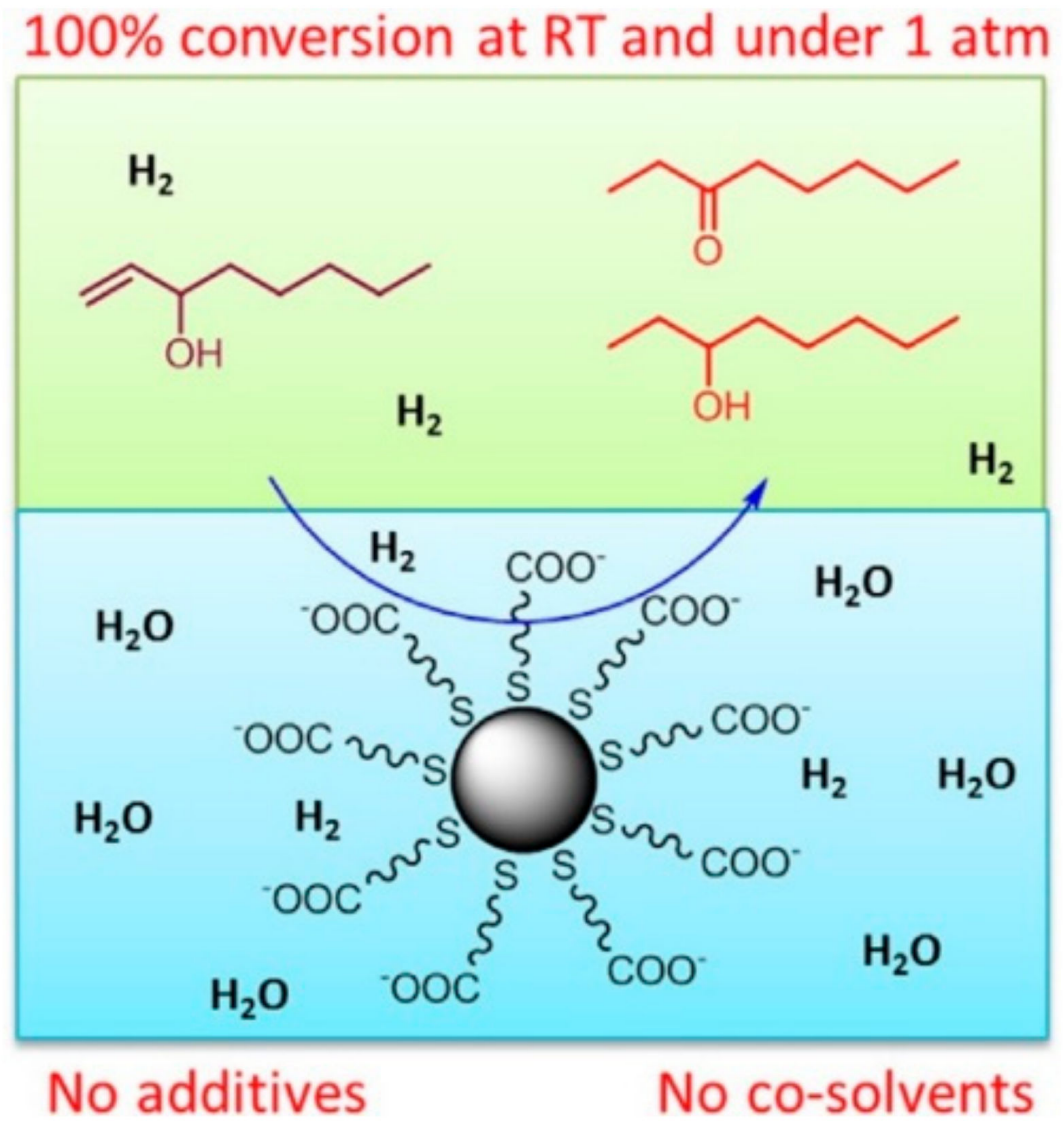
Biphasic catalytic reaction of hydrophobic allylic alcohols in water (5–10 mol % Pd/substrate, 50 μL substrate in 3 mL water or PBS buffer pH 7.4 solution, 1 atm H_2_). Reproduced from Ref. [72] with permission from the ELSEVIER, 2017.

Biphasic catalysis has also been performed using water-soluble PdNP with hydrophobic allylic alcohols (Figure 3b) [72]. Utilizing the biphasic reaction condition allowed the facile recycling of nanoparticles and simple product purifications. Catalysis studies showed that the activity and selectivity of the water-soluble PdNP strongly depend on the pH of the solvent. When the pH of the solvent was greater than 7, the PdNP was highly stable and soluble in aqueous media due to the presence of ionic carboxylate groups that can form electrostatic barriers.

**Scheme 8:**
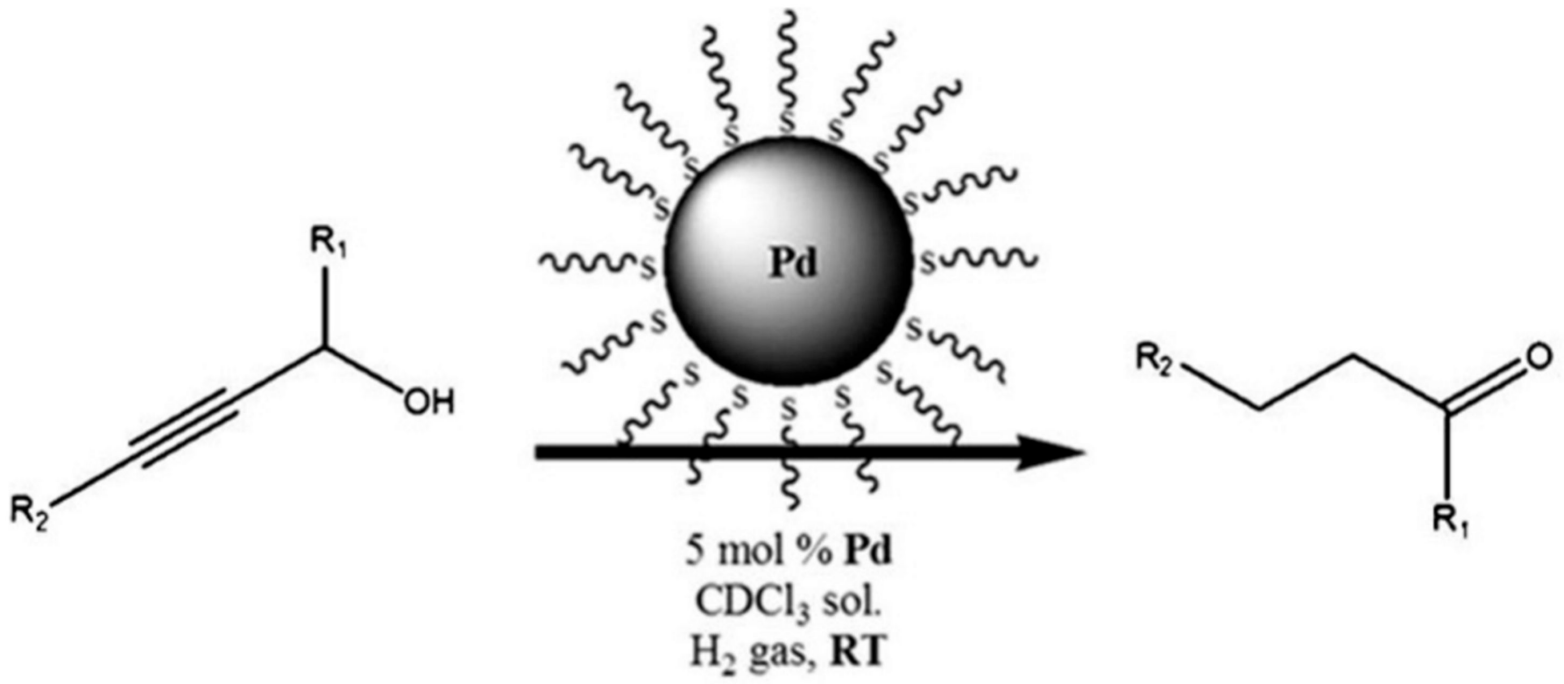
Semihydrogenation and isomerization of propargyl alcohols (5 mol % Pd/substrate, 50 μL substrate in 2 mL solvent, 1 atm H_2_). R_1-2_ = H or alkyl. Reproduced from Ref. [73] with permission from the Royal Society of Chemistry, 2013.

To take advantage of controlled poisoning, alkanethiolate-capped PdNP has been studied for the direct one-pot conversion of propargylic alcohols to their corresponding saturated carbonyls in homogenous condition (Scheme 8) [73]. Based on the kinetic studies, the reaction mechanism proceeded by first converting the substrate propargyl alcohol to the semihydrogenated product, allyl alcohol. Then, the intermediate allyl alcohol was mostly converted to isomerization product with the formation of some hydrogenation product. When an alkyl group presented at R_1_ position (Scheme 5), the reaction becomes even more selective toward the isomerization product due to the higher thermodynamic stability of the enol intermediate.

Alkanethiolate-capped PdNP has also been used for selective hydrogenation and/or isomerization of alkenes and dienes [74,75]. When pent-1-ene is subjected to unsupported, alkanethiolate-capped PdNP in CDCl_3_, the isomerization product (pent-2-ene) was generated as a predominant product (>90% selectivity) with only small amounts of pentane. Higher alkenes such as pent-1-ene are known to undergo hydrogenation with the formation of di-σ-bonded species on palladium surfaces [76]. The isomerization product (pent-2-ene) was generated via the mono-σ bonded Pd–alkyl intermediate followed by *β*-hydride elimination [77]. The poisoning of Pd surfaces by thiolate ligands prevented the alkene substrates from forming the strongly adsorbed di-σ-bonded species. Other higher alkenes, such as 3-phenylprop-1-ene and 4-phenylbut-1-ene, also underwent 92% conversion with 91% selectivity and >99% with 96% selectivity for isomerization, respectively. It was found that the hydrogenation of the terminal C=C bond can occur for alkanethiolate-capped PdNP by blocking isomerization, but it requires the presence of an activating group such as a phenyl ring. The presence of unhybridized p orbitals and planar geometry of the phenyl ring in a substrate like styrene would aid the formation of di-σ-bonded intermediate. In comparison, no hydrogenation reaction took place for 3,3-dimethylbut-1-ene. Di- and tri-substituted alkenes underwent hydrogenation very reluctantly by alkanethiolate-capped PdNP even with the presence of an activating phenyl group.

**Figure 4:**
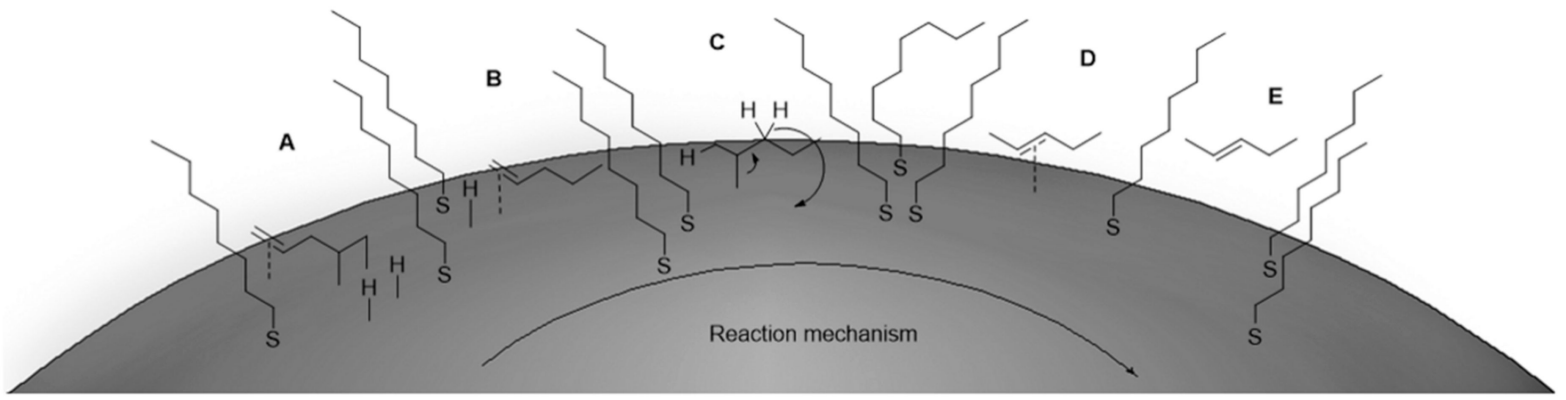
Proposed mechanism for the catalytic conversion of penta-1,4-diene by alkanethiolate-capped PdNP (5 mol % Pd/substrate, 0.5 mmol substrate in 2.5 mL CDCl_3_, 1 atm H_2_). Reproduced from Ref. [74] with permission from the Royal Society of Chemistry, 2015.

The catalytic reaction of penta-1,4-diene, an isolated diene, with alkanethiolate-capped PdNP produced pent-2-ene as a major product (Figure 4). The kinetic studies using ^1^H NMR revealed that the conversion first involved the direct di-σ-bond formation promoted via two Pd-π bond interactions (**A**). This is followed by the addition of two hydrogen atoms, resulting in the formation of pent-1-ene (**B**). With only a single alkene moiety, pent-1-ene isomerized to pent-2-ene via mono-σ bonded Pd–alkyl intermediate (**C**). This was followed by *β*-hydride reductive elimination to form a Pd-π bond intermediate (**D**) and pent-2-ene desorbed from the PdNP surface.

**Scheme 9:**
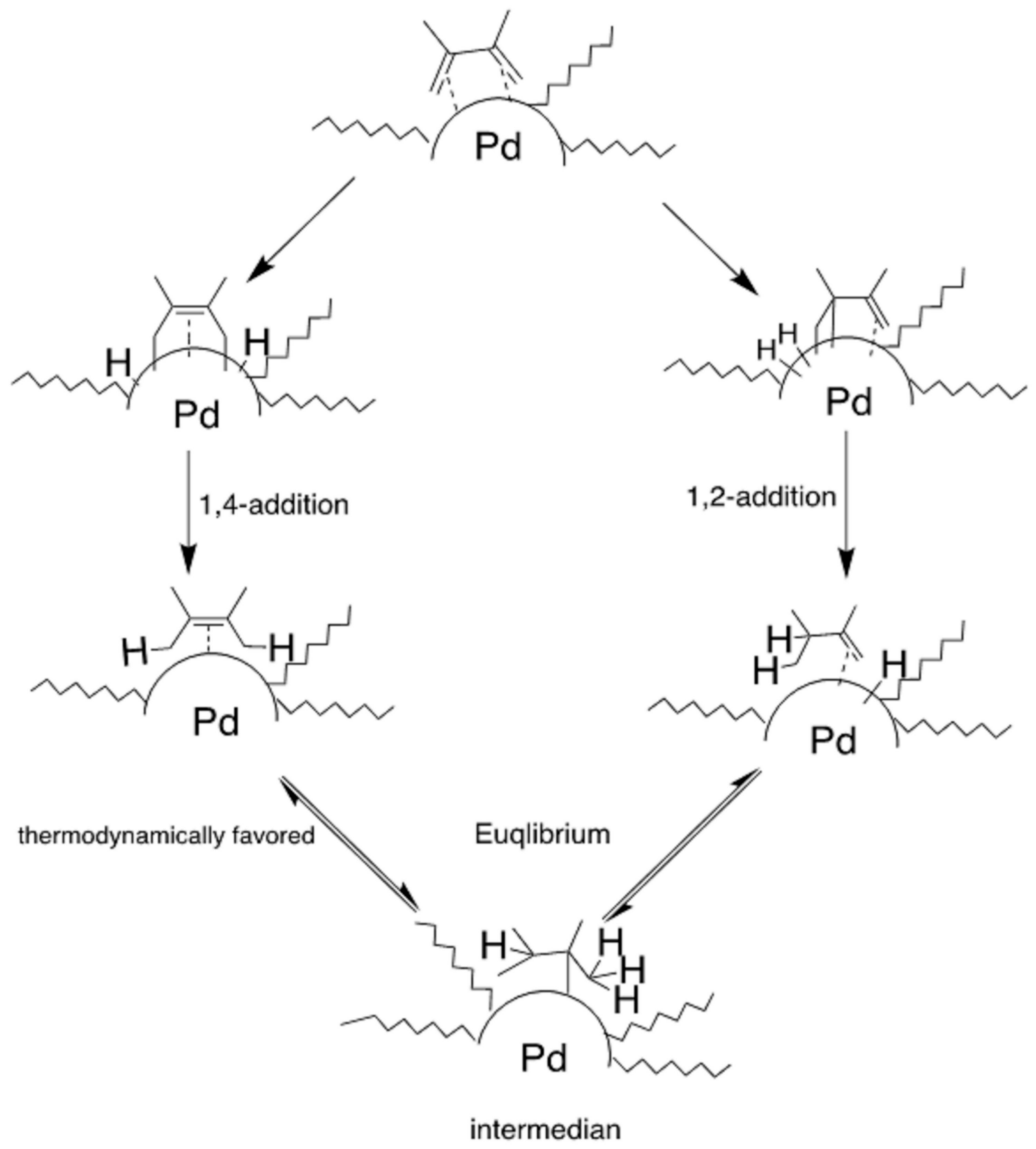
Proposed mechanism for the catalytic reaction of 2,3-dimethylbuta-1,3-diene with octanethiolate-capped Pd nanoparticles. Reproduced from Ref. [75] with permission from the Royal Society of Chemistry, 2017.

The catalytic reaction of alkanethiolate-capped PdNP with 2,3-dimethylbuta-1,3-diene exhibited an excellent selectivity toward the monohydrogenation products with 100% conversion yield [75]. The analysis of final monoene composition showed that the major product is 2,3-dimethylbut-2-ene (91%), the 1,4-addition product, and the minor product is 2,3-dimethylbut-1-ene (9%), the 1,2-addition product. The high yield of internal alkene was the result of both initial 1,4-addtion reaction and the subsequent isomerization of the terminal alkene, the 1,2-addition product, into the internal alkene shown in Scheme 9. Hydrogenation of dienes resulted in the higher 1,4-/1,2-addition ratio because the formation of 1,4-di-σ-bonded intermediate for these substrates was often sterically more accessible than that of the 1,2-di-σ-bonded intermediate. After 1 h of reaction, the conversion of 2,3-dimethylbuta-1,3-diene reached over 50%, with the ratio of 1,4-/1,2-addition products at 3.43. The consumption of 2,3-dimethylbuta-1,3-diene was almost complete after the 5 h reaction, with the ratio of 1,4-/1,2-addition products at 4.90. Further kinetic studies indicated that the yield of the 1,4-addtion product was still increasing after the 5 h reaction, with the ratio of 1,4-/1,2-addition products reaching to 10.30. This result corresponded well with the results observed for the isomerization of terminal alkenes to internal alkenes driven by the thermodynamic effect.

The selectivities for monohydrogenation and between the 1,4- and 1,2-addition products were also investigated for other diene and triene substrates as shown in Table 4. Alkanethiolate-capped PdNP clearly showed high selectivity for the 1,4-addition monohydrogenation product from the reactions of various dienes. PdNP was also highly selective toward the monohydrogenation of both ocimene (entry 6) and myrcene (entry 7). The presence of a large alkyl substituent group at the diene part of ocimene decreased the ratio of 1,4- and 1,2-addition products. The yield for the 1,2-addition internal alkene product was relatively high due to the good accessibility of the terminal alkene group onto Pd surface. Both major products in this case were internal alkenes resulting in the lack of thermodynamic driving force for isomerization. The ratio of 1,4-/1,2-addition products for the catalytic reaction of myrcene was, however, more than twice higher than that of ocimene. This was because the both terminal carbons of the diene group did not possess any substituent group subjecting to less steric interference for direct 1,4-hydrogen addition compared to ocimene. The thermodynamic stability of 1,4-addition internal alkene product was also higher than that of 1,2-addition product, making the isomerization more likely to occur.

### 3.2. Catalysis of Pt Nanoparticles

**Scheme 10:**
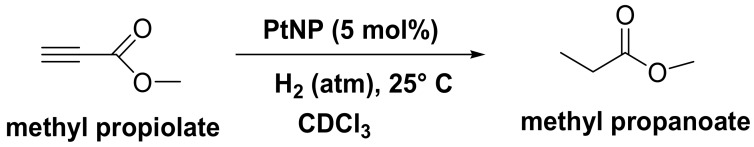
Catalysis reaction of methyl propiolate by PtNP (5 mol % Pt/substrate, 0.25 mmol substrate in 2 mL CDCl_3_, 1 atm H_2_). Reproduced from Ref. [64] with permission from the American Chemical Society, 2017.

Alkanethiolate-capped PtNP exhibited high activity for the hydrogenation of activated terminal alkynes [64]. The kinetic studies for the hydrogenation of methyl propionate indicated that the substrate is converted to 49% semihydrogenation product and 51% full-hydrogenated product after 3 h. At 6 h, the reaction was close to completion toward full hydrogenation, producing methyl propanoate (Scheme 10). The complete full-hydrogenation products were only obtained from the catalytic reactions of terminal alkynes with a conjugated carbonyl group such as methyl propiolate and 3-butyn-2-one. For the reaction of tert-butyl propiolate, PtNP produced the full-hydrogenation product in high yield (~95%), with ~5% of semihydrogenation product after 24 h. This indicates that the tert-butyl group of this substrate has a slight steric influence on the reactivity of PtNP. Unactivated alkynes without conjugation and bulkier internal alkynes would only result in low substrate conversions, whereas only trace amount of full-hydrogenation products was obtained. This chemoselectivity was due to the partial poisoning by alkanethiolate surface ligands, which clearly influences the geometric and electronic surface properties of colloidal PtNP. This unique selectivity of alkanethiolate-capped PtNP clearly implies the importance of developing a new synthetic protocol that allows the systematic partial poisoning of nanoparticle surfaces.

## 4. Conclusions and Perspective

The synthesis of metal nanoparticles has drawn a lot of attention due to their size, shape, and surface-state dependent properties in various applications such as catalysis. Organic ligands are often introduced to encapsulate the nanoparticles for better stability and solubility, but they are known to negatively impact their catalytic activity. This review, however, showed that it is possible to control the partial poisoning of nanoparticle catalysts using the thiosulfate protocol and turn them into selective colloidal catalytic materials. To expand the availability of selective catalysts for various organic reactions and develop a better understanding of critical structure–function relationships of ligand-capped nanoparticle catalysts, future research should continuously seek to develop methods for the synthesis of nanoparticle catalysts that have different ligand structures and functionality while balancing catalytic activity and stability.

Investigating the performance of small metal nanoparticle catalysts influenced by the presence of well-defined organic ligands resembling amino acid residues in an enzyme binding pocket can provide understanding of the effects of steric, noncovalent, and chiral interactions in the near-surface environment (or near-active site). Further advancements of this research using metal nanoparticle catalysts functionalized with well-defined small thiolate ligands with different hydrophobic or hydrophilic groups, therefore, can ultimately provide critical information regarding how nanocatalysts can tune catalytic selectivity precisely through specific substrate interactions. The availability of well-designed binary organic ligand-capped nanoparticles with an active catalytic core can also tremendously benefit the fundamental understanding of the substrate–receptor noncovalent interactions that operate in the binding pockets of enzymes. Genuine understanding on the effects of surface composition and distribution of binary thiolate ligands adsorbed on metal nanoparticle catalyst surfaces can allow us to distinguish the electronic and geometric contributions of the capping ligands on the catalytic activity and selectivity of nanoparticle catalysts.

The obtained knowledge of critical structure–function relationships can be applied to the development of optimized artificial nanocatalysts with high regioselectivity, chemoselectivity, and/or stereoselectivity, which has been one of the biggest challenges against further development of nanocatalysis. Besides their important roles in chemical industries and biology, catalysis also plays critical roles in health, environmental, and energy applications. The continued investigations of organic ligand-capped metal nanoparticle catalysts will likely have a large impact on the broad field of research in science and technology.

## Figures and Tables

**Table 1:** Reaction conditions for the synthesis of AuNPs with various core sizes. Data reproduced from Ref [60], 2010.

Reaction Conditions ^1^		Nanoparticles
MEEE BS/Au	T (°C)	d_core_ (nm)
1:100	60	21.4 ± ^2^
1:20	60	10.1 ± 3.6
1:6	24	6.1 ± 1.1
1:1	24	3.1 ± 1.1
3:1	24	1.6 ± 0.4

^1^ Molar equivalents of sodium borohydride with respect to metal precursor (10 to 1). ^2^ Greater than 50% polydispersity.

**Table 2:** Average core size of Ag nanoparticles. The ligand/silver molar ratio used for the synthesis is 1:1. Reproduced from Ref. [56], 2004.

Nanoparticles	TEM (Diameter, nm)
AgNP, Bunte	3.3 ± 0.8
AgNP, Bunte, 50 °C	3.5 ± 1.0
AgNP, thiol, Tol/H_2_O	3.4 ± 1.1
AgNP, Bunte, Tol/H_2_O	4.3 ± 1.0

**Table 3:** Different Synthetic Conditions and Characterization of Various PdNPs. The molar equivalent of each reaction species corresponds to one equivalent of Pd precursor. Data reproduced from Ref. [59], 2012.

Entry	Ligand	TOAB	NaBH_4_	T (°C)	TGA (% Pd)	TEM (Diameter, nm)	Approximate Average Molecular Formula	Ligand surf. Coverage (Ligands/Surface Atoms)
i	2	10	20	22	68.6	2.59 ± 1.15	~Pd_586_L_142_	0.52
ii	2	10	20	60	51.0	1.51 ± 0.46	~Pd_116_L_59_	0.75
iii	2	5	20	22	55.7	1.66 ± 0.62	~Pd_140_L_59_	0.61
iv	1	10	20	22	77.0	2.76 ± 1.15	~Pd_807_L_127_	0.37
v	1	10	5	22	80.6	3.38 ± 0.95	~Pd_1289_L_164_	0.34

**Table 4:** Selective hydrogenation of dienes and trienes by alkanethiolate-capped PdNP (5 mol % Pd/substrate, room temp., 1 atm H_2_). Data reproduced from Ref. [75], 2017.

Entry	Substrate	Reaction Condition	Major Product (%)	Percentage
1	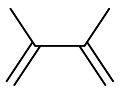	C8 PdNP, 24 h	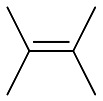	(91%) + (9% 1,2-)
2	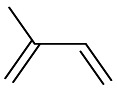	C8 PdNP, 24 h	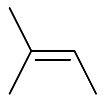	(93%) + (7% 1,2-)
3	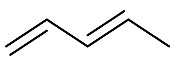	C8 PdNP, 24 h	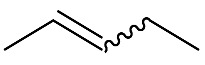	(92%) + (8% 1,2-)
4	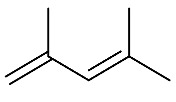	C8 PdNP, 24 h	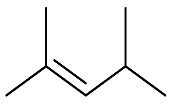	(90%) + (7% 1,2-)
5	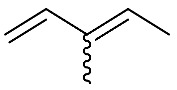	C8 PdNP, 24 h	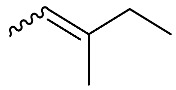	(90%) + (5% 1,2-)
6	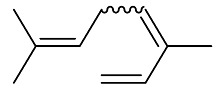	C8 PdNP, 24 h	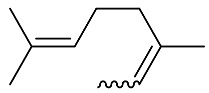	(59%) + (41% 1,2-)
7	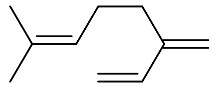	C8 PdNP, 24 h	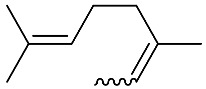	(69%) + (23% 1,2-)
